# Momentary Self-regulation: Scale Development and Preliminary Validation

**DOI:** 10.2196/35273

**Published:** 2022-05-10

**Authors:** Emily A Scherer, Sunny Jung Kim, Stephen A Metcalf, Mary Ann Sweeney, Jialing Wu, Haiyi Xie, Gina L Mazza, Matthew J Valente, David P MacKinnon, Lisa A Marsch

**Affiliations:** 1 Center for Technology and Behavioral Health Geisel School of Medicine at Dartmouth Lebanon, NH United States; 2 Department of Biomedical Data Science Geisel School of Medicine at Dartmouth Lebanon, NH United States; 3 Department of Health Behavior and Policy School of Medicine Virginia Commonwealth University Richmond, VA United States; 4 Health Communication and Digital Innovation Massey Cancer Center Virginia Commonwealth University Richmond, VA United States; 5 Department of Public Health and Primary Care University of Cambridge Cambridge United Kingdom; 6 School of Media and Design Shanghai JiaoTong University Shanghai China; 7 Department of Computer Science Dartmouth College Hanover, NH United States; 8 Department of Psychology Arizona State University Tempe, AZ United States; 9 Department of Quantitative Health Sciences Mayo Clinic Scottsdale, AZ United States; 10 Center for Children and Families Department of Psychology Florida International University Miami, FL United States

**Keywords:** self-regulation, momentary self-regulation, ecological momentary assessment, psychometric, health behavior change, health risk behaviors, mobile phone

## Abstract

**Background:**

Self-regulation refers to a person’s ability to manage their cognitive, emotional, and behavioral processes to achieve long-term goals. Most prior research has examined self-regulation at the individual level; however, individual-level assessments do not allow the examination of dynamic patterns of intraindividual variability in self-regulation and thus cannot aid in understanding potential malleable processes of self-regulation that may occur in response to the daily environment.

**Objective:**

This study aims to develop a brief, psychometrically sound momentary self-regulation scale that can be practically administered through participants’ mobile devices at a momentary level.

**Methods:**

This study was conducted in 2 phases. In the first phase, in a sample of 522 adults collected as part of a larger self-regulation project, we examined 23 previously validated assessments of self-regulation containing 594 items in total to evaluate the underlying structure of self-regulation via exploratory and confirmatory factor analyses. We then selected 20 trait-level items to be carried forward to the second phase. In the second phase, we converted each item into a momentary question and piloted the momentary items in a sample of 53 adults over 14 days. Using the results from the momentary pilot study, we explored the psychometric properties of the items and assessed their underlying structure. We then proposed a set of subscale and total score calculations.

**Results:**

In the first phase, the selected individual-level items appeared to measure 4 factors of self-regulation. The factors identified were perseverance, sensation seeking, emotion regulation, and mindfulness. In the second phase of the ecological momentary assessment pilot, the selected items demonstrated strong construct validity as well as predictive validity for health risk behaviors.

**Conclusions:**

Our findings provide preliminary evidence for a 12-item momentary self-regulation scale comprising 4 subscales designed to capture self-regulatory dynamics at the momentary level.

## Introduction

### Background

Self-regulation refers to a person’s ability to manage emotions, cognition, and behavior to avoid immediate gratification, which may interfere with achieving long-term goals. People with self-regulatory competence tend to make goal-oriented decisions and inhibit impulsive behavior that is incompatible with their long-term goals [1]. Self-regulation lapses are linked to social and health problems, such as poor academic outcomes, obesity, substance use disorders, and preventable deaths [2-4].

An individual’s level of self-regulation is likely responsive to internal factors, such as negative affect or stress, which may lessen an individual’s determination or available resources to focus on self-regulatory behavior, as well as external environmental factors, such as being in a location with many temptations for risk behaviors. However, published measures of self-regulation are typically based on retrospective self-reports obtained through cross-sectional surveys or task-based methods. As in other assessments of psychological constructs (eg, self-esteem and personality), retrospective self-report methods collected at 1 time point are helpful in measuring dispositional states, allowing researchers to quantitatively describe an individual and examine interindividual variability, but are limited in measuring the changing states of such constructs within an individual [5,6]. Task-based assessments have similarly been developed for single or paired assessments before and after a defined stimulus and are typically delivered in a laboratory setting. Thus, traditional trait-level assessments may fall short of examining dynamic patterns reflecting intraindividual variability in self-regulation and understanding malleable processes of one’s self-regulation in a naturalistic setting (as aligned with the contextual model of self-regulation proposed by Roos and Witkiewitz [7]).

A methodologically and psychometrically sound metric that precisely and sensitively captures malleable processes involved in self-regulation in a real-world setting may enable a more contextually informed understanding of self-regulatory processes. Developing a valid assay for measuring changes in self-regulation in a nonlaboratory, everyday setting may help researchers better identify the construct’s responsiveness to internal and environmental factors and thus more effectively intervene in self-regulation as a putative mechanism that may play a causal role in facilitating health behavior change. Proliferation in information and communication technologies, combined with novel measurement methods, such as smartphone-based ecological momentary assessment (EMA), enables researchers to examine and assess self-regulatory processes and dynamics at a momentary level as people move through their lives in various real-world environmental contexts (ie, in contrast to laboratory settings or retrospective recall).

### Overall Study Objective

#### Overview

This study aimed to develop a brief, psychometrically sound momentary self-regulation scale that can be practically administered through participants’ mobile devices at a momentary level. The objective was for the momentary scale to capture the constructs measured in existing self-regulation measures and capture both intra- and interindividual variability in self-regulation as it occurs in naturalistic settings. This work is part of a broader exploration of the ontology of self-regulation supported by the National Institutes of Health’s Science of Behavior Change initiative [8]. The work was conducted in 2 distinct phases.

#### Phase 1: Measuring Self-regulation at the Individual Level Using Existing Scale Items

Beginning with a broad representation of items putatively measuring self-regulation at the individual level, we aimed to understand the underlying dimensions measured by these items and then select a smaller subset of items that capture these dimensions well and that could be studied at the moment level in a naturalistic setting.

#### Phase 2: Scale Development and Preliminary Validation

Beginning with the items selected in phase 1, we aimed to modify the items for measurement at the moment level, pilot their use via momentary assessment methods, construct a momentary scale, and preliminarily assess its psychometric properties at the moment level.

## Methods

### Phase 1

#### Overview

Starting with a comprehensive set of existing scales that purport to measure self-regulation at the individual level, we aimed to confirm each scale’s factor structure and characterize its item characteristics. We then assessed the underlying constructs measured by the full set of items from all scales and determined their factor structure. Considering this as the underlying structure of self-regulation, we made an initial selection of the best-performing items from each factor. We aimed to confirm that the factor structure of self-regulation was preserved when using this limited set of items. Once we finalized a set of items that performed well and together measured all factors identified as part of the constructs measured at the individual level, we moved to study them further at the moment level in phase 2.

#### Literature Review and Scale and Task Selection

The larger self-regulation initiative began with a comprehensive review of the scientific literature of assessments (both survey based and cognitive task based) used in the domain of self-regulation research and related constructs (eg, impulsivity, mindfulness, behavioral disinhibition, and temporal discounting). This review outlined the origin of all assays and the conceptual and empirical associations between the data from each measure and health and social behaviors. This study identified 23 self-report surveys and 37 cognitive tasks that purport to measure some aspect of self-regulation. The process for selecting the scales has been described in detail elsewhere [9,10]. Briefly, these scales were chosen for their ability to measure underlying latent constructs of the umbrella construct of *self-regulation*. Self-regulation refers to a person’s ability to manage cognitive, motivational, and emotional resources to act in accordance with their long-term goals. The constructs were operationalized as cognitive functions that allow an individual to engage in effective self-regulatory behaviors. Measures that focused on aspects of self-regulation such as goal planning, self-regulation failures, impulsivity, cognitive control, and temporal discounting were sampled.

#### Sample and Sample Partitioning

Next, a sample of 522 adults was recruited through Amazon Mechanical Turk (MTurk), a crowdsourcing website, and the 594 items from the 23 surveys were administered. A subset of these individuals (150/522, 28.7%) was selected to complete the surveys again 3 months later to enable the assessment of test-retest reliability [11]. A description of the MTurk study design and sample recruitment procedures is described elsewhere, as is a description of the participants in the sample and their scores on the surveys and behavioral tasks [8,9]. For this project, survey data from this sample were used to perform a dimension analysis of a range of self-regulation measures. A full list of the surveys and their subscales is included in Multimedia Appendix 1 [6,12-31].

To support the dimension analysis via exploratory factor analysis (EFA), the observations (participants) were partitioned into a discovery set (200/522, 38.3%) and a validation set (322/522, 61.7%). The complete set (N=522) comprised the discovery and validation sets.

#### Analytic Approach

##### Item Reduction to Solve an n<p Problem

In statistics, an *n<p* problem describes the challenge of having more variables than observations on which the variables are measured. With the MTurk sample of 522, we encountered such a challenge in using all the variables (items) to perform a dimension analysis of self-regulation using all 594 items from the 23 self-regulation surveys. Therefore, a precursor to performing exploratory factor analyses was the reduction of the number of evaluated items while still maintaining items in each potential self-regulation domain. To facilitate this process, scale-level analyses were performed on each self-reported scale to first confirm the structure of the scale and then identify a subset of *well-performing* items to carry forward as candidates for further self-regulation dimension analysis. This process began with research on the scale’s derivation and a qualitative review of the scale to ensure self-regulation was indeed the scale’s target. Next, for each scale, correlated factor analysis was performed to confirm the scale’s factor structure in the sample. Finally, an item response theory (IRT) analysis was performed within each scale to identify a set of approximately 3 items per scale to inform the development of a measure that succinctly captures various dimensions of self-regulation. Items were considered to perform well in the factor analysis if they loaded primarily on one factor of the scale and did not have high loadings on other factors and were considered to perform well in the IRT analysis if they had high information and discrimination. The goal of the item reduction process was to keep items from each subscale or construct measured to retain full coverage of scales in the final candidate set while limiting them to well-performing items.

##### Dimension Analysis and Factor Interpretation

The MTurk data on the reduced set of items (116 items) were subjected to dimensional analyses. The discovery (n=200), validation (n=322), and complete data sets (N=522) were used to perform the EFA of the reduced set of items. The goal of the EFA was to identify the number of underlying factors in the sample and interpret the content of each factor where possible. EFA was performed using Mplus [32].

For each factor-based solution, a qualitative review of the results was performed to identify and describe the factors. This assessment was done by focusing on items that loaded onto a factor and then qualitatively reviewing the text of these items and naming the factor based on the content of all loading items.

##### Test-Retest Results

Test-retest information from each item was also considered in the item selection process. In the sample of 150 MTurk participants who completed the surveys at 2 time points, we computed item-level intraclass correlation coefficients (ICCs). The results of the scale and task ICCs and how they related to behaviors have been published elsewhere [11]. For this study, the ICCs provided further information on which items might be better for momentary measurement. A large ICC indicates that there is not a great deal of variability within individuals. Such an item likely measures an individual-level characteristic rather than a momentary characteristic that may vary over time and in different situations. Details on how the ICCs were incorporated into the selection of items for study at a momentary level are included in the item selection process described in the following sections.

##### Item Selection for Study of Momentary Self-regulation

Using the results of the dimension analyses, we aimed to select a set of items from each identified factor and select approximately 15 items in total. The goal for the number of items selected for the momentary study was based on the number thought to be reasonable to answer on a momentary basis (consistent with the broader literature on EMA), as well as provide a large enough sample of items so that further item selection could be performed based on their performance in the planned pilot of the items (phase 2). To obtain a set of items from each factor, we selected items with the following characteristics:

1. Items that loaded primarily on one factor (did not load >0.5 on >1 factor) and loaded highly on that factor (>0.5)
2. Items whose ICC was not large enough to indicate a lack of variability within the individual
3. Items that did not refer to a specific activity that would not be present in a large proportion of moments in a real-world setting (eg, skiing or skydiving)

##### Confirming the Factor Structure

Once the items were selected for further study at the momentary level, we performed a confirmatory factor analysis (CFA) to determine whether the identified factors in the larger set of items were confirmed in the selected items. Adjustments to the item selection and further exploratory analyses and CFAs were performed until a solution with acceptable fit statistics was obtained.

### Phase 2

#### Overview

Using the items selected in phase 1, we moved to study self-regulation at a momentary level. We piloted all selected items on a new sample of participants and then assessed within- and between-individual variability of items and examined the underlying factor structure at the within- and between-individual levels. The goal was to develop a momentary self-regulation measure and perform an initial evaluation of its validity. To ensure construct validity between the trait-level and momentary-level self-regulation measurements, we examined the association between nonmomentary self-regulation measured at baseline and the momentary self-regulation responses collected from phase 2 participants throughout a total of 42 time points over 14 consecutive days. We also assessed the psychometric properties of between- and within-individual momentary self-regulation items and subscales, as well as the predictive validity of the subscales and total scores for health risk behaviors (eg, smoking and overeating). Self-regulation has been implicated in many health risk behaviors, including substance use and disordered eating [33-37].

#### Sample

A new sample of participants was recruited through MTurk for phase 2. To be eligible for the momentary study, participants had to be aged between 18 and 50 years, US residents in states that included only the Eastern time zone (because of the manual process involved in implementing the text prompting and compensation procedures), fluent in English, and willing to receive text prompts on their smartphone to initiate and complete 3 surveys per day over 14 consecutive days. We recruited 60 participants to account for potential study attrition and meet a minimum analytic sample size of 50. We did not perform a formal power analysis or sample size calculation, given the exploratory nature of the study and the lack of preexisting data on the variability of momentary self-regulation. However, we expected that the sample size (n=50), along with 42 time points for each participant, would provide a rich data set for performing the proposed psychometric analyses.

Eligible participants were provided with information on the study procedure, risks, benefits, and payment schedules. Individuals who read the information sheet and agreed to participate enrolled in the study after clicking the *next* button on the page to provide their consent. They were directed to a web-based baseline survey that assessed demographic characteristics and several trait-level self-regulation surveys. Participants received study instructions and detailed explanations via phone SMS text messages and direct messages sent through the MTurk crowdsourcing platform.

#### Ethics Approval

The study procedure and survey materials were approved by the Dartmouth College Committee for the Protection of Human Subjects (STUDY00028975).

#### Data Collection

We leveraged the technology features available through mobile texting prompts and the Qualtrics survey platform to simulate EMA methods with 42 repeated microsurveys (3 times per day—morning, midday, and evening—for 14 days) and facilitate rapid compensation. Each microsurvey contained 20 survey items and took <3 minutes on average to complete. Participants were compensated within an hour of the completion of each microsurvey. We simulated the EMA method instead of developing an EMA mobile app to expedite data collection while enabling remote recruitment and data collection. Each text prompt contained a unique hyperlink that directed the participants to a given web-based microsurvey. Text prompts were sent at a random time within a predetermined time window. Participants were asked to complete microsurveys on their mobile devices (verified via an external website that tracks the devices used to answer surveys). To promote study retention, we sent a reminder with the same message to those who did not complete the survey within an hour of receiving the random prompt. Consistent with compensation models offered within the MTurk crowdsourcing environment, participants were compensated US $0.30 for each microassessment and US $5 daily bonuses for completing all 3 assessments per day.

#### Measures

##### Baseline Survey

We measured demographic characteristics (eg, age, gender, ethnicity, race, education, and income) and height and weight for BMI, as well as health behavior characteristics, such as the Drug Abuse Screening Test (DAST-10) [38,39], Cannabis Use Disorders Identification Test–Revised (CUDIT-R) [40], Alcohol Use Disorders Identification Test (AUDIT) [41], Three-Factor Eating Questionnaire-R18 (TFEQ-R18) [42], and smoking status. These behavioral characteristics were collected to examine the relationship between momentary self-regulation dynamics and health risk behaviors. In phase 1, a subset of items from the 23 self-report surveys was selected. In the phase 2 baseline survey, we included the 8 self-report surveys from which the momentary self-regulation scale items were selected. These scales were the functional and dysfunctional impulsivity subscales from the Dickman Impulsivity Inventory [12]; the suppression subscale of the Emotion Regulation Questionnaire (ERQ) [13]; the nonjudging subscale of the Five Facet Mindfulness Questionnaire (FFMQ) [14]; the venturesomeness subscale of the Eysenck I-7 Impulsiveness and Venturesomeness Questionnaire (I-7) [15]; the Mindful Attention Awareness Scale (MAAS) [16]; the Selection, Optimization, and Compensation Questionnaire [17]; the Short Self-Regulation Questionnaire (SSRQ) [18]; and the lack of premeditation and lack of perseverance subscales of the Urgency, Premeditation (lack of), Perseverance (lack of), Sensation Seeking, and Positive Urgency (UPPS-P) Impulsive Behavior Scale [19,20]. These subscales were collected to facilitate validation analyses for the momentary items.

##### Microsurveys

Driven by the practice of momentary scale development and validation research in psychometric studies [43], the wording of the 20 items was modified to capture the momentary level of self-regulation. For example, the item “I keep my emotions to myself” was modified to “Since the last prompt, I kept my emotions to myself.” Similar modification methods with the leading phrase “Since the last prompt...” were applied to all other candidate items. Response options for all items were standardized to a 5-point Likert scale, ranging from *not at all* (1) to *extremely* (5). After phase 1 and data collection for phase 2, we noted that 2 of the 20 items originated from the Multidimensional Personality Questionnaire Control scale [21], which has a copyright restriction that prevents their use in the development and publishing of new measures. Therefore, we removed these 2 items from the originally selected items, verified that the other 22 self-regulation surveys did not have copyright restrictions, and proceeded with 18 items in the analysis. As detailed in the following sections, the 18 items did not have a different structure than that of the 20 items.

#### Analytic Approach

First, we evaluated the construct validity of the 20 individual momentary items using baseline self-regulation surveys. To do this, we fit generalized estimating equation models to examine the association between each momentary self-regulation item and its corresponding trait-level self-regulation subscale assessed at baseline. This was to ensure what we intended to measure at a momentary level (self-regulation at the moment of each assessment) was the same concept (self-regulation) captured by the original self-regulation survey.

Second, to assess the intra- and interindividual variability of the items, we examined ICCs estimated via univariate multilevel models with a probit link. We then performed multilevel EFA followed by CFA, allowing for correlated factors to identify the number of factors measured by the momentary set of items at the between- and within-individual levels. The results of these factor analyses, along with the information on construct validity and ICCs, were used to select approximately 3 items per factor to be included in the final momentary scale.

With the final set of momentary self-regulation items, we created subscale scores comprising the mean item response from all items from a factor and created overall scores computed as the mean of the 4 subscale scores. We evaluated the construct validity of the final momentary subscales and total scores via mixed-effects models examining the relationship between the momentary subscale and total score and the baseline self-regulation measures. Finally, we evaluated the predictive validity using mixed-effects models for health information (eg, alcohol, smoking, other substance use, food intake, and BMI) and explored the association between momentary self-regulation subscales and age, sex, education, and income. All analyses accommodated the multilevel structure of the data by modeling both the between- and within-individual variations in repeated assessments. Mplus [32] was used for the multilevel factor analyses. SAS software (version 9.4, SAS Institute Inc) was used for data merging and processing, generalized estimating equations, and mixed models.

## Results

### Phase 1

#### Item Reduction

Owing to space constraints, we do not present the results from each scale-level analysis used to select a reduced set of well-performing items. Instead, we briefly describe the steps taken for the UPPS-P Impulsive Behavior Scale as an example. The same process was followed for all 23 self-regulation surveys.

First, the 5-factor structure was confirmed through a factor analysis of all the items on the scale. The factors loaded onto the subscales that were previously defined in the literature. The selected items loaded strongly onto their designated factors and showed a minimal overlap. Separate scree plots for each subscale confirmed the 1-factor structure of the subscales.

The selected items from each subscale are described in Textbox 1. These items were selected based on a qualitative assessment to identify the items with the best discrimination and highest level of information.

**Selected items from each subscale of the Urgency, Premeditation (lack of), Perseverance (lack of), Sensation Seeking, and Positive Urgency Impulsive Behavior Scale.**

**Selected items from each subscale**

**Premeditation**

- “I like to stop and think things over before I do them.” [UPP17]
- “I usually think carefully before doing anything.” [UPP49]
- “Before making up my mind, I consider all the advantages and disadvantages.” [UPP56]

**Perseverance**

- “I generally like to see things through to the end.” [UPP05]
- “I finish what I start.” [UPP28]
- “I almost always finish projects that I start.” [UPP43]

**Negative urgency**

- “When I feel bad, I will often do things I later regret in order to make myself feel better now.” [UPP18]
- “When I am upset I often act without thinking.” [UPP30]
- “I often make matters worse because I act without thinking when I am upset.” [UPP45]

**Positive urgency**

- “When I get really happy about something, I tend to do things that can have bad consequences.” [UPP41]
- “When overjoyed, I feel like I can’t stop myself from going overboard.” [UPP46]
- “I tend to act without thinking when I am really excited.” [UPP53]

**Sensation seeking**

- “I quite enjoy taking risks.” [UPP24]
- “I welcome new and exciting experiences and sensations, even if they are a little frightening and unconventional.” [UPP32]
- “I sometimes like doing things that are a bit frightening.” [UPP42]

#### Dimension Analysis and Factor Interpretation

##### Number of Factors: EFA

Scree plots of eigenvalues from the discovery, validation, and complete sets (reduced items) showed a decreased rate of change after 3 eigenvalues and were almost flat after 6 eigenvalues. Given this, 3- and 4-factor EFAs were completed.

##### Interpretation of Factors

Patterns of factor loadings were used to interpret the measured factors across several factor solutions. For each solution, all items loading on a factor were reviewed, and an attempt was made to identify the construct under study and assign a name to each factor through a qualitative assessment of the item text. Table 1 lists the names of the factors across multiple factor-based solutions.

In the 3-factor solution, in both the discovery and validation sets, as well as the complete observation set, the factors appeared to represent (1) perseverance or lack of impulsivity, (2) sensation seeking, and (3) inhibition or mind over matter. In the 4-factor solution, in both the discovery and validation sets, the factors appeared to represent (1) perseverance, (2) impulsivity or sensation seeking, (3) inhibition or mind over matter, and (4) negative rumination or self-judgment. However, in the complete observation set, the factors differed, appearing to represent (1) impulsivity, (2) sensation seeking, (3) inhibition or mind over matter, and (4) negative rumination or self-judgment. Overall, the 3-factor solution had the most support based on the scree plot, consistency of results across observation sets, and additional exploratory factor analyses not presented. Therefore, we moved to the next step, assuming a 3-factor solution.

**Table 1:** Phase 1: factor interpretation for the 3- and 4-factor exploratory factor analysis.

		Factor analysis and data set						
		3-factor				4-factor		
		Discovery	Validation	Complete	Discovery		Validation	Complete
**Factor interpretation**								
	Perseverance or lack of impulsivity	✓	✓	✓				
	Perseverance				✓		✓	
	Sensation seeking	✓	✓	✓				✓
	Impulsivity or sensation seeking				✓		✓	
	Inhibition or mind over matter	✓	✓	✓	✓		✓	✓
	Impulsivity							✓
	Negative rumination or self-judgment				✓		✓	✓

#### Item Selection for Study of Momentary Self-regulation

As 3 factors appeared to have the most support, and among the 3-factor solutions, the discovery, validation, and complete data sets yielded similar factors and results, we considered the complete set and 3-factor solution as the results from which we would identify items for further study at the momentary level. The selection process is detailed in Textbox 2.

**Item selection process.**

**Item selection process based on the complete data set and 3-factor solution**

1. From each factor, we removed items with factor loadings <0.5 or with factor loadings >0.5 on >1 factor from further consideration.
2. Among the remaining items, the perseverance factor had many more items with factor loadings >0.5 (49 items), whereas the other 2 factors (sensation seeking and inhibition or mind over matter) each had a smaller number of items with loadings >0.5 (15 and 9 items, respectively). As we wanted to represent all 3 factors in our selected items, we only considered the first 21 items (approximately 40% of all items with loadings >0.5) that loaded onto the perseverance factor (ordered by factor loading value). For sensation seeking and inhibition or mind over matter, we evaluated all items with loadings >0.5 onto the factor.
3. Among the resultant 21+15+9 items, we aimed to select items in proportion to the number loading on the 3 factors; hence, we sought to select 10 perseverance or impulsivity items, 3 sensation-seeking items, and 2 inhibition or mind over matter items. The selection procedure was as follows:
  We removed items with a large intraclass correlation coefficient (ICC) value (>0.7).
  We removed items referring to a specific activity (eg, skiing or skydiving), which is common among sensation-seeking items.
  We added items outside of the initial selection from each factor if they narrowly missed the selection but had available ICCs that were <0.4.
  We then selected items that represented a variety of themes within the factor, favoring items with lower ICCs.

The selection resulted in 20 initially selected items: 10 items from the perseverance or impulsivity factor, 5 items from the sensation-seeking factor, and 5 items from the inhibition or mind over matter factor. Given that the items that appeared to represent *impulsivity* (eg, “I think before doing,” “do you generally do and say things without stopping to think?” and “I get in trouble because I don’t think before I act”) were not selected, we call the first factor *perseverance* rather than *perseverance or lack of impulsivity*.

#### Confirming the Factor Structure

We performed a CFA to determine whether the identified factors in the larger set of items were confirmed in the selected 20 items. The 3-factor confirmatory model did not fit the data well (root mean square error of approximation [RMSEA] 0.115, 95% CI 0.110-0.121; Tucker-Lewis Index [TLI] 0.801). Therefore, we performed a 3-, 4-, and 5-factor EFA of the selected items. In the exploratory analysis, the 4-factor solution best fit the data (RMSEA 0.051, 95% CI 0.043-0.059; TLI 0.961). Qualitative examination of items that had previously made up the third factor (inhibition or mind over matter) showed a split across the third and fourth factors in the EFA, suggesting 2 separate factors: emotion regulation and mindfulness. To accommodate this new structure, from the originally selected 20 items, 2 items were removed from the perseverance factor, and 1 item each was selected (based on the qualitative assessment of measurement and examination of factor loadings within the larger item set and item-level ICCs) to measure the emotion regulation and mindfulness factors. Furthermore, a 4-factor CFA was performed on the revised 20-item set, and the model fit the data well (RMSEA 0.094, 95% CI 0.088-0.100; TLI 0.917), and we considered our selection complete. Tables 2 and 3 show the items initially selected and the final item selection, respectively, that resulted from the confirmatory analyses. The factors identified were perseverance, sensation seeking, emotion regulation, and mindfulness.

**Table 2:** Phase 1: initial (before confirmatory analysis) selection of items that represent 3 underlying factors to be considered candidates for momentary measurement in phase 2.

Item source	Item text	Factor name		
		Perseverance	Sensation seeking	Inhibition or mind over matter
UPPS-P^a^ Impulsive Behavior Scale [19,20]	I finish what I start.	✓		
Selection, Optimization, and Compensation Questionnaire [17]	I keep working on what I have planned until I succeed. When I do not succeed right away at what I want to do I do not try other possibilities for very long.	✓		
UPPS-P Impulsive Behavior Scale [19,20]	I generally like to see things through to the end.	✓		
Short Self-Regulation Survey [18]	I set goals for myself and keep track of my progress.	✓		
Dickman Impulsivity Inventory [12]	I often say and do things without considering the consequences.	✓		
UPPS-P Impulsive Behavior Scale [19,20]	I usually think carefully before doing anything.	✓		
Multidimensional Personality Questionnaire [21]	I am careful in reasoning.	✓		
10-Item Personality Questionnaire [6]	Dependable, self-disciplined.	✓		
Multidimensional Personality Questionnaire [21]	I value a rational approach.	✓		
Short Self-Regulation Survey [18]	I am able to resist temptation.	✓		
Eysenck I-7 Impulsiveness and Venturesomeness Questionnaire [15]	Do you quite enjoy taking risks?		✓	
Eysenck I-7 Impulsiveness and Venturesomeness Questionnaire [15]	Do you sometimes like doing things that are a bit frightening?		✓	
Dickman Impulsivity Inventory [12]	I am good at taking advantage of unexpected opportunities, where you have to do something immediately or lose your chance.		✓	
UPPS-P Impulsive Behavior Scale [19,20]	I have a reserved and cautious attitude toward life.		✓	
Dickman Impulsivity Inventory [12]	I like to take part in really fast-paced conversations, where you don’t have much time to think before you speak.		✓	
Five Facet Mindfulness Questionnaire [14]	I think some of my emotions are bad or inappropriate and I shouldn’t feel them.			✓
Emotion Regulation Questionnaire [13]	When I am feeling negative emotions, I make sure not to express them.			✓
Emotion Regulation Questionnaire [13]	I control my emotions by not expressing them.			✓
Mindful Attention Awareness Scale [16]	I find myself doing things without paying attention.			✓
Mindful Attention Awareness Scale [16]	It seems I am “running on automatic” without much awareness of what I’m doing.			✓

^a^UPPS-P: Urgency, Premeditation (lack of), Perseverance (lack of), Sensation Seeking, and Positive Urgency.

**Table 3:** Phase 1: final (after confirmatory analysis) selection of items that will be considered candidates for momentary measurement in phase 2.

Factor name	Item text	Factor name			
		Perseverance	Sensation seeking	Emotion regulation	Mindfulness
UPPS-P^a^ Impulsive Behavior Scale [19,20]	I finish what I start.	✓			
Selection, Optimization, and Compensation Questionnaire [17]	I keep working on what I have planned until I succeed. When I do not succeed right away at what I want to do I do not try other possibilities for very long.	✓			
UPPS-P Impulsive Behavior Scale [19,20]	I generally like to see things through to the end.	✓			
Short Self-Regulation Survey [18]	I set goals for myself and keep track of my progress.	✓			
Dickman Impulsivity Inventory [12]	I often say and do things without considering the consequences.	✓			
UPPS-P Impulsive Behavior Scale [19,20]	I usually think carefully before doing anything.	✓			
Multidimensional Personality Questionnaire [21]	I am careful in reasoning.	✓			
Multidimensional Personality Questionnaire [21]	I value a rational approach.	✓			
Short Self-Regulation Survey [18]	I am able to resist temptation.	✓			
Eysenck I-7 Impulsiveness and Venturesomeness Questionnaire [15]	Do you quite enjoy taking risks?		✓		
Eysenck I-7 Impulsiveness and Venturesomeness Questionnaire [15]	Do you sometimes like doing things that are a bit frightening?		✓		
Dickman Impulsivity Inventory [12]	I am good at taking advantage of unexpected opportunities, where you have to do something immediately or lose your chance.		✓		
UPPS-P Impulsive Behavior Scale [19,20]	I have a reserved and cautious attitude toward life.		✓		
Five Facet Mindfulness Questionnaire [14]	I think some of my emotions are bad or inappropriate and I shouldn’t feel them.			✓	
Emotion Regulation Questionnaire [13]	When I am feeling negative emotions, I make sure not to express them.			✓	
Emotion Regulation Questionnaire [13]	I control my emotions by not expressing them.			✓	
Five Facet Mindfulness Questionnaire [14]	I tell myself I shouldn’t be thinking the way I am thinking.			✓	
Mindful Attention Awareness Scale [16]	I find myself doing things without paying attention.				✓
Mindful Attention Awareness Scale [16]	It seems I am “running on automatic” without much awareness of what I’m doing.				✓
Mindful Attention Awareness Scale [16]	I rush through activities without being really attentive to them.				✓

^a^UPPS-P: Urgency, Premeditation (lack of), Perseverance (lack of), Sensation Seeking, and Positive Urgency.

### Phase 2

#### Participants and Study Retention

Approximately 12% (7/60) of participants who enrolled in the study did not complete any of the 42 microsurveys, resulting in 53 participants with momentary data. Participants were mainly White (47/53, 88%), non-Hispanic (48/53, 90%), married (20/53, 37%), and female (30/53, 56%) and aged between 22 and 48 years at baseline. These 53 participants completed a median of 42 of the 42 microsurveys (range 1-42). The mean BMI was 27.18 (SD 6.08, range 18.30-44.30) kg/m^2^, falling in the overweight category. Approximately 30% (16/53) of the participants reported that they smoked cigarettes every day, 40% (21/53) had used cannabis, and 36% (19/53) reported that they had not limited their alcohol intake to less than monthly in the past year. Approximately 28% (15/53) of participants reported that they had used drugs other than those required for medical reasons.

#### Momentary Item Examinations: Construct Validity and Intra- and Interindividual Variability

Analyses examining the construct validity of the individual items assessed the association between the baseline trait-level self-regulation item and momentary-level self-regulation item. They showed a significant association with the exception of 1 item (item 3), indicating that for all other items, the concept operationalized and measured through momentary items reflects the original construct (trait-level self-regulation) measured at the baseline survey (Table 4).

Unconditional multilevel models confirmed that there was significant variation at the individual item level for all items with ICCs ranging from 37% to 64%, indicating that a large proportion of the variability in the items was because of within-individual fluctuation rather than between-individual variation.

**Table 4:** Phase 2 construct validity of momentary items: regression coefficients from multilevel models relating momentary self-regulation items to corresponding baseline trait-level self-regulation subscales.

Item number	Source (baseline self-regulation scale)	Momentary self-regulation item	Regression coefficient
1	Dickman Impulsivity Inventory [12]	Since the last prompt, I’ve been good at taking advantage of unexpected opportunities.	0.65^a^
2	Dickman Impulsivity Inventory [12]	Since the last prompt, I’ve said things without considering the consequences.	0.56^a^
3	Emotion Regulation Questionnaire [13]	Since the last prompt, I’ve controlled my emotions by not expressing them.	0.10
4	Emotion Regulation Questionnaire [13]	Since the last prompt, if I’ve felt negative emotions, I’ve made sure not to express them.	0.19^a^
5	Five Facet Mindfulness Questionnaire [14]	Since the last prompt, I’ve told myself I shouldn’t be thinking the way I am thinking.	0.28^b^
6	Five Facet Mindfulness Questionnaire [14]	Since the last prompt, I’ve thought some of my emotions are bad or inappropriate and I shouldn’t feel them.	0.21^b^
7	Eysenck I-7 Impulsiveness and Venturesomeness Questionnaire [15]	Since the last prompt, I’ve quite enjoyed taking risks.	0.89^a^
8	Eysenck I-7 Impulsiveness and Venturesomeness Questionnaire [15]	Since the last prompt, I’ve enjoyed doing things that are a bit frightening.	0.50^a^
9	Mindful Attention Awareness Scale [16]	Since the last prompt, it has seemed I am running on “automatic” without much awareness of what I’m doing.	−0.44^a^
10	Mindful Attention Awareness Scale [16]	Since the last prompt, I’ve rushed through activities without really being attentive to them.	−0.42^a^
11	Mindful Attention Awareness Scale [16]	Since the last prompt, I’ve found myself doing things without paying attention.	−0.43^a^
14	Selection, Optimization, and Compensation Questionnaire [17]	Since the last prompt, I’ve worked on what I planned until I succeeded.	−1.05^a^
15	Short Self-Regulation Questionnaire [18]	Since the last prompt, I have set goals and kept track of my progress toward goals.	0.49^a^
16	Short Self-Regulation Questionnaire [18]	Since the last prompt, I’ve been able to resist temptation.	0.29^a^
17	UPPS-P^c^ Impulsive Behavior Scale [19,20]	Since the last prompt, my attitude toward life has been reserved and cautious.	−0.47^a^
18	UPPS-P Impulsive Behavior Scale [19,20]	Since the last prompt, I’ve generally seen things through to the end.	−0.68^a^
19	UPPS-P Impulsive Behavior Scale [19,20]	Since the last prompt, I’ve been able to finish projects I started.	−0.43^a^
20	UPPS-P Impulsive Behavior Scale [19,20]	Since the last prompt, I have thought carefully before doing things.	−0.61^a^

^a^*P*≤.01.

^b^*P*≤.05.

^c^UPPS-P: Urgency, Premeditation (lack of), Perseverance (lack of), Sensation Seeking, and Positive Urgency.

#### Examining Factor Structure in Momentary Items

Multilevel EFA models with between 3 and 5 within-individual factors and between 3 and 4 between-individual factors were explored. Table 5 presents the fit statistics for these models. Models with both 4 and 5 within-individual factors and with both 3 and 4 between-individual factors had good fit statistics, with the exception of the chi-square test, which is sensitive to sample size.

We used iterative processes to report the best factor solutions for the momentary self-regulation scale. For example, although the 5-factor within-individual structure seemed to fit the data well, having 5 factors resulted in separating the emotion regulation factor into 2 factors based only on the scales from which the items originated, which is unlikely to be a meaningful distinction. The 3-factor solution at the between-individual level appeared to fit the data well. The difference between the 3- and 4-factor solutions is that emotion regulation and mindfulness factors may be combined into 1 factor at the between-individual level. Given these results, we examined the 4-factor EFA results for the item selection steps that followed. The 4 factors identified were very similar to those present in phase 1: perseverance, sensation seeking, mindfulness, and emotion regulation.

As we aimed to retain only items that performed well at the individual and moment levels in a momentary self-regulation scale, we examined the factor loadings and communalities of the items in the multilevel EFA. We selected items that consistently showed high communalities at both within- and between-individual levels, indicating that variability in these items is reasonably explained by the underlying factors, as well as items that consistently loaded highly on the factors of interest and did not simultaneously load highly on the other factors being measured. This process yielded a set of 12 items—3 selected for each underlying factor.

**Figure 1:**
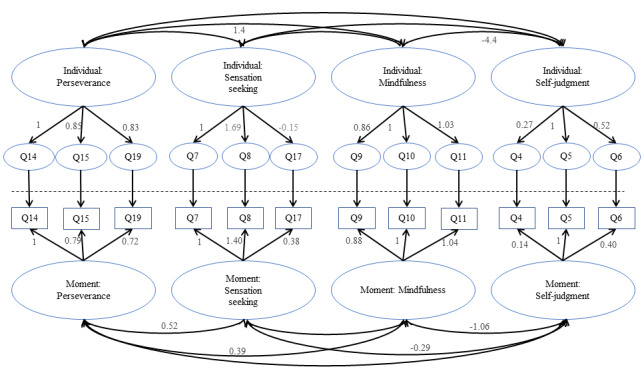
Path coefficients from the multilevel confirmatory factor analysis of 12 finalized items. For path coefficients between latent factors, only significant coefficients are reported for readability.

A multilevel CFA was fit to these 12 items. The fit statistics from this multilevel CFA model were comparative fit index 0.989, standardized root mean squared residual (between) 0.083, standardized root mean square residual (within) 0.083, RMSEA 0.012, and TLI 0.985. The significant path coefficients and covariances between the factors are shown in Figure 1.

**Table 5:** Phase 2 fit statistics from multilevel exploratory factor analyses on 18 momentary items.

Statistics	Exploratory factor analysis model				
	3W3B^a^	4W3B^b^	5W3B^c^	4W4B^d^	5W4B^e^
Number of parameters	192	207	221	222	236
Chi-square (*df*)^f^	606.7 (204)^f^	322.6 (189)^f^	222.7 (175)^f^	317.6 (174)^f^	213.1 (160)^f^
RMSEA^g^	0.030	0.018	0.011	0.020	0.012
CFI^h^	0.913	0.971	0.990	0.969	0.988
TLI^i^	0.869	0.953	0.982	0.945	0.978
SRMR^j^ within	0.130	0.091	0.065	0.091	0.065
SRMR between	0.064	0.064	0.064	0.042	0.042

^a^Model with 3 within-level factors and 3 between-level factors.

^b^Model with 4 within-level factors and 3 between-level factors.

^c^Model with 5 within-level factors and 3 between-level factors.

^d^Model with 4 within-level factors and 4 between-level factors.

^e^Model with 5 within-level factors and 4 between-level factors.

^f^*P*<.01.

^g^RMSEA: root mean square error of approximation.

^h^CFI: comparative fit index.

^i^TLI: Tucker-Lewis Index.

^j^SRMR: standardized root mean square residual.

#### Subscale and Total Score Creation

To create subscale scores based on the 4 identified factors, we calculated the mean of the scores of all items loading onto a factor and reverse coded the items so that all items measured the construct in the same direction. For example, the sensation-seeking subscale comprised the mean of items 7 and 8, which measure sensation-seeking behavior, and reverse-coded item 17, which measures a lack of sensation-seeking behavior. In addition, we reverse coded all mindfulness items (items 9, 10, and 11) to measure mindfulness rather than lack of mindfulness.

We also created a total momentary self-regulation scale score that was a composite (mean value) of all subscale scores using 2 methods: literature-based total score and data-based total score. On the basis of the existing literature, we would expect that perseverance, mindfulness, emotion regulation, and lack of sensation-seeking factors would all have positive correlations. Therefore, we created a literature-based total by calculating the mean of the perseverance, mindfulness, emotion regulation, and reverse-coded sensation-seeking subscales (literature-based total).

In the CFA (and EFAs), we found that the emotion regulation and perseverance factors had a negative correlation. Therefore, we examined the items that make up the emotion regulation factor, and instead, they appeared to measure self-judgment to some extent. Indeed, the items were from the nonjudging scale of the FFMQ. Self-judgment could be negative rather than positive in terms of self-regulation; thus, we created another total score comprising the mean of the perseverance, mindfulness, reverse-coded emotion regulation, and reverse-coded sensation-seeking subscales (data-based total). Higher literature-based and data-based totals each indicate greater momentary self-regulation. Table 6 includes the final item set and details on how each item contributes to the subscale and how the subscales contribute to the total score.

**Table 6:** Phase 2 momentary-level items in momentary self-regulation scale.

Item number	Source	Momentary self-regulation item	Momentary self-regulation subscale (-)^a^ (R)^b^
14	Selection, Optimization, and Compensation Questionnaire [17]	Since the last prompt, I’ve worked on what I planned until I succeeded.	Perseverance
15	Short Self-Regulation Questionnaire [18]	Since the last prompt, I have set goals and kept track of my progress toward goals.	Perseverance
19	UPPS-P^c^ Impulsive Behavior Scale [19,20]	Since the last prompt, I’ve been able to finish projects I started.	Perseverance
7	Eysenck I-7 Impulsiveness and Venturesomeness Questionnaire [15]	Since the last prompt, I’ve quite enjoyed taking risks.	(-) Sensation seeking
8	Eysenck I-7 Impulsiveness and Venturesomeness Questionnaire [15]	Since the last prompt, I’ve enjoyed doing things that are a bit frightening.	(-) Sensation seeking
17	UPPS-P Impulsive Behavior Scale [19,20]	Since the last prompt, my attitude towards life has been reserved and cautious.	(-) Sensation seeking (R)
4	Emotion Regulation Questionnaire [13]	Since the last prompt, if I’ve felt negative emotions, I’ve made sure not to express them.	(-) Self-judgment
5	Five Facet Mindfulness Questionnaire [14]	Since the last prompt, I’ve told myself I shouldn’t be thinking the way I am thinking.	(-) Self-judgment
6	Five Facet Mindfulness Questionnaire [14]	Since the last prompt, I’ve thought some of my emotions are bad or inappropriate and I shouldn’t feel them.	(-) Self-judgment
9	Mindful Attention Awareness Scale [16]	Since the last prompt, it has seemed I am running on “automatic” without much awareness of what I’m doing.	Mindfulness (R)
10	Mindful Attention Awareness Scale [16]	Since the last prompt, I’ve rushed through activities without really being attentive to them.	Mindfulness (R)
11	Mindful Attention Awareness Scale [16]	Since the last prompt, I’ve found myself doing things without paying attention.	Mindfulness (R)

^a^Indicates the subscale should be reverse coded when combining to create the data-based total score.

^b^Indicates the item should be reverse coded when creating the subscale score.

^c^UPPS-P: Urgency, Premeditation (lack of), Perseverance (lack of), Sensation Seeking, and Positive Urgency.

#### Construct Validity of Momentary Subscales and Total Scores

We examined the relationships between the momentary self-regulation subscales and the trait-level self-regulation subscales by modeling the associations between the momentary subscales and the literature-based and data-based total scores with the trait-level subscale scores assessed at baseline. The results are presented in Table 7. A regression coefficient that is significantly different from 0 would support construct validity, as we expected the momentary subscales to relate to existing scales that measure self-regulation. The strength of the association varied across the baseline measures and momentary subscales. This may be because of different subscales measuring different aspects of self-regulation than the particular baseline measure, or it may be because of a greater degree of individual variability in the momentary scale. The data-based total was associated with the suppression subscale of the ERQ, the nonjudging subscale of the FFMQ, the MAAS, the SSRQ, and the lack of premeditation and lack of perseverance subscales of the UPPS-P. The literature-based total was associated with the venturesomeness subscale of the I-7, the MAAS, the SSRQ, and the lack of premeditation and lack of perseverance subscales of the UPPS-P.

The momentary mindfulness subscale was significantly associated with the trait-level suppression subscale of the ERQ, the nonjudging subscale of the FFMQ, the venturesomeness subscale of the I-7 (marginal, *P*=.05), the MAAS, the SSRQ, and the lack of premeditation and lack of perseverance subscales in the UPPS-P, indicating various convergent features of the momentary mindfulness subscale with trait-level surveys.

Momentary emotion regulation or self-judgment demonstrated a significant association with the suppression subscale of the ERQ and a marginally significant association with the nonjudging subscale of the FFMQ, indicating the convergent properties of the momentary self-judgment subscale. The momentary perseverance subscale was related to the MAAS, the SSRQ, and the lack of perseverance and lack of premeditation subscales of the UPPS-P. The momentary sensation-seeking subscale was associated with the functional impulsivity subscale of the Dickman Impulsivity Inventory, the venturesomeness subscale of the I-7, and the lack of premeditation subscale of the UPPS-P.

**Table 7:** Phase 2 regression coefficients from multilevel models relating baseline trait-level existing self-regulation subscales with momentary self-regulation subscales and total scores (based on 12 items).

Trait-level scale (subscale, if applicable)	Momentary-level subscale					Momentary-level scale	
	Mindfulness	Self-judgment	Perseverance	Sensation seeking	Data-based total^a^		Literature-based total^b^
Dickman Impulsivity Inventory (functional impulsivity) [12]	−0.21	−0.17	0.37	0.48^c^	−0.02		−0.12
Dickman Impulsivity Inventory (dysfunctional impulsivity) [12]	−0.29	0.27	−0.21	0.32	−0.28		−0.13
Emotion Regulation Questionnaire (suppression) [13]	−0.10^c^	0.08^c^	−0.01	0.01	–0.05^d^		−0.01
Five Facet Mindfulness Questionnaire (nonjudging) [14]	−0.15^c^	0.09	−0.08	−0.01	−0.09^c^		−0.03
Eysenck I-7 Impulsiveness and Venturesomeness Questionnaire (venturesomeness) [15]	–0.25^d^	0.02	0.14	0.39^c^	−0.13		–0.12^d^
Mindful Attention Awareness Scale [16]	0.17^c^	−0.03	0.11^c^	−0.02	0.08^c^		0.07^c^
Short Self-Regulation Questionnaire [18]	0.12^d^	0.03	0.24^c^	0.01	0.09^c^		0.25^c^
UPPS-P^e^ Impulsive Behavior Scale (lack of premeditation) [19,20]	−0.20^d^	0.01	–0.19^d^	0.23^c^	−0.17^c^		0.10^c^
UPPS-P Impulsive Behavior Scale (lack of perseverance) [19,20]	–0.17^d^	0.02	−0.33^c^	0.00	−0.13^c^		−0.15^c^

^a^Data-based total comprising the mean of mindfulness, perseverance, reverse-coded self-judgment, and reverse-coded sensation seeking.

^b^Literature-based total comprising the mean of mindfulness, perseverance, self-judgment, and reverse-coded sensation seeking.

^c^*P*≤.01.

^d^*P*≤.05.

^e^UPPS-P: Urgency, Premeditation (lack of), Perseverance (lack of), Sensation Seeking, and Positive Urgency.

#### Predictive Validity

The associations between the subscales and the total scores and behavioral characteristic variables are presented in Table 8. The mindfulness subscale was significantly negatively associated with the TFEQ-R18 total, TFEQ-R18 uncontrolled eating, TFEQ-R18 emotional eating, and having never smoked and positively associated with age, such that older individuals had higher scores on momentary mindfulness. The momentary self-judgment subscale was significantly positively associated with the DAST-10 total, TFEQ-R18 total, TFEQ-R18 emotional eating, and TFEQ-R18 cognitive restraint.

**Table 8:** Regression coefficients from multilevel models relating demographics and health behaviors to momentary self-regulation subscales and total scores.

Demographic and health behavior measures		Mindfulness	Self-judgment	Perseverance	Sensation seeking	Data-based total^a^	Literature-based total^b^
AUDIT^c^ total		−0.03	0.03	0	0.06^d^	–0.03^d^	−0.02
**AUDIT categories**							
	1	1.14	−0.9	0.23	–1.14^d^	0.9^d^	0.43
	2	1.29^e^	−1.04	0.42	−0.72	0.92^d^	0.37
	3	−0.18	0.25	0.24	0.03	0	0.04
	4 (reference)	0	0	0	0	0	0
CUDIT-R^f^ total		−0.06	0.05	0.02	≥0.06	−0.04	−0.01
DAST-10^g^ total		−0.09	0.09^e^	0.06	0.11^d^	−0.06	−0.02
TFEQ-R18^h^ total		–0.03^d^	0.03^d^	−0.01	0.003	–0.02^d^	0.00
TFEQ-R18 uncontrolled eating		–0.01^e^	0.004	−0.01	−0.0004	–0.01^e^	−0.004
TFEQ-R18 emotional eating		–0.01^e^	0.01^e^	−0.01	0.001	–0.005^d^	−0.002
TFEQ cognitive restraint		−0.003	0.01^d^	0.003	0.003	−0.004	0.003
**Smoking status**							
	Current	−0.36	0.19	−0.26	0.25	−0.27	−0.14
	Never	–0.61^e^	0.30	−0.39	0.09	–0.35^e^	−0.21
	Past (reference)	0	0	0	0	0	—
Age (years)		0.03^e^	−0.02	−0.003	–0.03^e^	0.02^e^	0.01
Sex (male)		−0.22	0.08	0.19	0.31	−0.11	−0.05
BMI (kg/m^2^)		−0.02	−0.01	−0.02	−0.01	−0.01	0.01
**Education**							
	Bachelor’s degree	−0.15	0.36	−0.27	0.28	−0.27	−0.05
	High School or GED^i^	−0.07	0.08	−0.25	0.22	−0.16	0.01
	Master’s degree	−0.27	0.48	0.33	0.09	−0.13	−0.05
	Some college (reference)	0	0	0	0	0	0
**Income (US $)**							
	<30,000	0.02	0.16	−0.23	−0.01	−0.09	−0.06
	30,000-50,000	−0.24	0.08	–0.98^d^	0.41	–0.44^e^	–0.41^e^
	50,000-70,000	0.01	0.05	–0.63^d^	−0.05	−0.16	−0.16
	>70,000 (reference)	0	0	0	0	0	0

^a^Data-based total comprises the means of mindfulness, perseverance, reverse-coded self-judgment, and reverse-coded sensation seeking.

^b^Literature-based total comprises the means of mindfulness, perseverance, self-judgment, and reverse-coded sensation seeking.

^c^AUDIT: Alcohol Use Disorders Identification Test.

^d^*P*≤.01.

^e^*P*≤.05.

^f^CUDIT-R: Cannabis Use Disorders Identification Test–Revised.

^g^DAST-10: Drug Abuse Screening Test.

^h^TFEQ-R18: Three-Factor Eating Questionnaire-R18 (revised version with 18 questions).

^i^GED: General Educational Development.

The momentary perseverance subscale was negatively associated with only specific income categories; perseverance scores tended to decrease among people with an income ranging between US $30,000 and US $70,000. The momentary sensation-seeking subscale was significantly positively associated with the AUDIT, CUDIT-R, and DAST-10 total scores and negatively associated with age.

The data-based total score (mean of perseverance, mindfulness, reverse-coded self-judgment, and reverse-coded sensation seeking) had the most associations with negative health behaviors and is therefore considered the optimal way of combining the subscales into a total score for the predictive ability for risk behaviors. This data-based total score was significantly negatively associated with the AUDIT total, TFEQ-R18 total, TFEQ-R18 uncontrolled eating, TFEQ-R18 emotional eating, and having never smoked and positively associated with low categories of the AUDIT. The data-based total also had a significant association with older age. Although the pairwise comparison between those in the US $30,000 to US $50,000 income category and those in the ≥US $70,000 income category had significantly different data-based total scores, income was not significantly associated with the data-based total score overall. In contrast, the literature-based total (mean of perseverance, mindfulness, emotion regulation, and reverse-coded sensation seeking) was not related to any negative health behaviors. The literature-based total had a significant association with income (type 3 *P* value=.04), with those in the US $30,000 to US $50,000 income category reporting a significantly lower literature-based total score than those in the ≥US $70,000 income category.

## Discussion

### Principal Findings

We developed and tested a momentary self-regulation scale, starting with a broad literature review on the overarching concept of self-regulation. Using an empirically driven, iterative data analytic and refinement process with 23 self-regulation-related surveys, in phase 1 we conducted dimension-level and item-level analyses and reduction through EFA, CFA, and IRT to select the best candidate set of 20 survey items with which to develop the momentary self-regulation scale. The phase 1 results indicated that existing self-regulation scales measure several underlying constructs within self-regulation, including perseverance, sensation seeking, emotion regulation, and mindfulness. We demonstrated that these constructs could be measured at the individual level using a reduced set of items from these scales. In phase 2, we examined the factor structure, item loadings, and within- and between-individual variations to further reduce the 20 candidate items to 4 subscales with 3 items each. The final 12-item momentary self-regulation scale totals and subscales demonstrated construct validity relative to the trait-level scales from which they were derived, as well as predictive validity for health risk behaviors. The phase 2 results provide initial evidence that momentary self-regulation varies both at the individual level and within individuals across time in a real-world setting. The resulting metric may be useful for assessing factors that promote or fail to promote self-regulation, including within-individual variation in self-regulation. It may also be useful in assessing when interventions do or do not promote self-regulation, a putative mechanism of behavior change in many populations.

Research using EMA has illuminated processes that drive not only time-sensitive, environment-responsive health behaviors, such as substance use [44], but also psychological functioning, such as impulsivity [43]. Digital technology has enabled this research so that psychological and behavioral processes can be explored within and between individuals as well as within a nonlaboratory, naturalistic setting. A granular understanding of self-regulation as a mechanism of behavior change is key to understanding the conditions under which health behavior change interventions may produce replicable effects and inform the development and refinement of more effective behavior change interventions. To facilitate this understanding, new measures of known individual characteristics that can be captured on a momentary basis are needed. The current work demonstrates that self-regulation is one construct that can be explored at this level, as it varies both within and between individuals. These findings also indicate that this momentary self-regulation scale can be administered through mobile devices in a naturalistic setting. This scale may be useful in capturing the richness of self-regulatory function in vivo and in changing contexts and may help further inform contextual models of self-regulation.

### Limitations

Although the final factor structure and items used in the momentary self-regulation scale have demonstrated evidence for construct validity and predictive validity, as well as intraindividual level variations, this study has several limitations. First, both samples were drawn from a population of MTurk workers whose representativeness is unknown relative to the broader US population. Basic demographic information indicated that the sample had limited racial and ethnic diversity. Future studies should examine whether these findings can be replicated and extended to other populations. Furthermore, the momentary (phase 2) study did not measure environmental contextual factors or social interactions of participants, which may have affected the responses. Future efforts may adapt the study methods and analytic procedures to capture these external factors as momentary self-regulatory dynamics are likely to be interactive with, and reactive to, environmental and internal factors.

In addition, in phase 2, we recruited a sample of 60 individuals who resided in states in the Eastern time zone in this study because of the operational capacity of the research team and the manual process involved in sending text prompts with microsurveys at randomized times. Although we do not have reason to believe that persons from states in the Eastern time zone have significantly different self-regulatory dynamics and characteristics than those in other areas, future studies should examine the validity and reliability of our scale among individuals recruited from geographically diverse areas and groups, including rural and urban areas.

### Conclusions

Beginning with existing self-regulation scales, we examined the underlying constructs measured by the full set of items in the scales and then identified a reduced set of items that captured the underlying constructs measured across the scales. After confirming that the construct structure was retained within the reduced set of items, we further piloted the set of items in a momentary study in which we captured data from individuals in the moment, in their naturalistic contexts. Using the momentary study results, we further reduced the item set and demonstrated the initial validity in this sample of a momentary self-regulation scale comprising 12 items spanning 4 momentary self-regulation subscales. To further evaluate the validity and reliability more generally, the momentary self-regulation scale should be evaluated in other samples and contexts. This novel momentary self-regulation scale measures self-regulation on a momentary basis as individuals move through their daily lives and can be administered on mobile devices.

## Appendix

Multimedia Appendix 1 Self-report surveys list.

## Abbreviations

AUDIT: Alcohol Use Disorders Identification Test
CFA: confirmatory factor analysis
CUDIT-R: Cannabis Use Disorders Identification Test–Revised
DAST-10: Drug Abuse Screening Test
EFA: exploratory factor analysis
EMA: ecological momentary assessment
ERQ: Emotion Regulation Questionnaire
FFMQ: Five Facet Mindfulness Questionnaire
I-7: Eysenck I-7 Impulsiveness and Venturesomeness Questionnaire
ICC: intraclass correlation coefficient
IRT: item response theory
MAAS: Mindful Attention Awareness Scale
MTurk: Amazon Mechanical Turk
RMSEA: root mean square error of approximation
SSRQ: Short Self-Regulation Questionnaire
TFEQ-R18: Three-Factor Eating Questionnaire-R18 (revised version with 18 questions)
TLI: Tucker-Lewis Index
UPPS-P: Urgency, Premeditation (lack of), Perseverance (lack of), Sensation Seeking, and Positive Urgency
