# Supplementary material for: Momentary Self-regulation: Scale Development and Preliminary Validation
Source: JMIR Ment Health. 2022 May 10;9(5):e35273. doi: 10.2196/35273 (PMC9131140; doi:10.2196/35273)
Supplement: Multimedia Appendix 1 [file mental_v9i5e35273_app1.docx]

**Self-Report Surveys List**

| **Self-Report Surveys (abbreviation)** | **Subscales** | **References** |
| --- | --- | --- |
| Barratt Impulsivity Scale | Attentional | [22] |
|  | Motor |  |
|  | Non-Planning |  |
| Behavioral Avoidance/Inhibition Scales | BAS Drive | [23] |
|  | BAS Fun-Seeking |  |
|  | BAS Reward-Responsiveness |  |
|  | BIS |  |
| Brief Self-Control Scale | Self-Control | [24] |
| Dickman Impulsivity Inventory | Dysfunctional | [12] |
|  | Functional |  |
| Domain-Specific Risk-Taking Scale  (Expected Benefits/  Risk Perception/  Risk Taking) | Ethical | [25] |
|  | Financial |  |
|  | Health/Safety |  |
|  | Recreational |  |
|  | Social |  |
| Emotion Regulation Questionnaire (ERQ) | Reappraisal | [13] |
|  | Suppression |  |
| Five Facet Mindfulness Questionnaire (FFMQ) | Acts with Awareness | [14] |
|  | Describe |  |
|  | Non-Judging |  |
|  | Non-Reactive |  |
|  | Observe |  |
| Eysenck I-7 Impulsive & Venturesome Survey (I-7) | Impulsiveness | [15] |
|  | Venturesomeness |  |
| Future Time Perspective | Future-Time Perspective | [26] |
| Mindful Attention Awareness Scale (MAAS) | Mindfulness | [16] |
| Multidimensional Personality Questionnaire (MPQ)  (Control subscale) | Control | [21] |
| Selection, Optimization, and Compensation Questionnaire (SOC) | Elective Selection | [17] |
|  | Loss-based Selection |  |
|  | Compensation |  |
|  | Optimization |  |
| Sensation Seeking Survey V | Boredom Susceptibility | [27] |
|  | Disinhibition |  |
|  | Experience Seeking |  |
|  | Thrill/Adventure Seeking |  |
| Short Grit Scale | Grit | [28] |
| Short Self-Regulation Survey (SSRQ) | Control | [18] |
| Stanford Leisure-Time Activity Categorical Item | Activity Level | [29] |
| Ten-Item Personality Questionnaire | Agreeableness | [6] |
|  | Conscientiousness |  |
|  | Emotional Stability |  |
|  | Extraversion |  |
|  | Openness |  |
| Theories of Willpower Scale | Endorse Limited Resource | [30] |
| Three-Factor Eating Questionnaire (TFEQ-R18) | Cognitive Restraint | [42] |
|  | Emotional Eating |  |
|  | Uncontrolled Eating |  |
| Urgency, Premeditation (lack of), Perseverance (lack of), Sensation Seeing, Positive Urgency, Impulsive Behavior Scale (UPPS-P) | Lack of Perseverance | [19, 20] |
|  | Lack of Premeditation |  |
|  | Negative Urgency |  |
|  | Positive Urgency |  |
|  | Sensation Seeking |  |
| Zimbardo Time Perspective Survey | Future | [31] |
|  | Past Negative |  |
|  | Past Positive |  |
|  | Present Fatalistic |  |
|  | Present Hedonistic |  |
